# Allelic expression of *AhNSP2-B07* due to parent of origin affects peanut nodulation

**DOI:** 10.3389/fpls.2023.1193465

**Published:** 2023-06-22

**Authors:** Zifan Zhao, Yichun Wang, Ze Peng, Ziliang Luo, Meixia Zhao, Jianping Wang

**Affiliations:** ^1^ Agronomy Department, University of Florida, Gainesville, FL, United States; ^2^ Plant Molecular and Cellular Biology Program, University of Florida, Gainesville, FL, United States; ^3^ College of Horticulture, South China Agricultural University, Guangzhou, China; ^4^ Department of Microbiology and Cell Science, Institute of Food and Agricultural Sciences, University of Florida, Gainesville, FL, United States

**Keywords:** non-Mendelian inheritance, CAPS marker, monoallelic expression, parent of origin, genomic imprinting, DNA methylation

## Abstract

Legumes are well-known for establishing a symbiotic relationship with rhizobia in root nodules to fix nitrogen from the atmosphere. The nodulation signaling pathway 2 (*NSP2*) gene plays a critical role in the symbiotic signaling pathway. In cultivated peanut, an allotetraploid (2n = 4x = 40, AABB) legume crop, natural polymorphisms in a pair of *NSP2* homoeologs (*N_a_* and *N_b_*) located on chromosomes A08 and B07, respectively, can cause loss of nodulation. Interestingly, some heterozygous (*N_B_n_b_*) progeny produced nodules, while some others do not, suggesting non-Mendelian inheritance in the segregating population at the N_b_ locus. In this study, we investigated the non-Mendelian inheritance at the *N_B_* locus. Selfing populations were developed to validate the genotypical and phenotypical segregating ratios. Allelic expression was detected in roots, ovaries, and pollens of heterozygous plants. Bisulfite PCR and sequencing of the *N_b_* gene in gametic tissue were performed to detect the DNA methylation variations of this gene in different gametic tissues. The results showed that only one allele at the Nb locus expressed in peanut roots during symbiosis. In the heterozygous (*N_b_n_b_*) plants, if dominant allele expressed, the plants produced nodules, if recessive allele expressed, then no nodules were produced. qRT-PCR experiments revealed that the expression of *N_b_* gene in the ovary was extremely low, about seven times lower than that in pollen, regardless of genotypes or phenotypes of the plants at this locus. The results indicated that *N_b_* gene expression in peanut depends on the parent of origin and is imprinted in female gametes. However, no significant differences of DNA methylation level were detected between these two gametic tissues by bisulfite PCR and sequencing. The results suggested that the remarkable low expression of *N_b_* in female gametes may not be caused by DNA methylation. This study provided a unique genetic basis of a key gene involved in peanut symbiosis, which could facilitate understanding the regulation of gene expression in symbiosis in polyploid legumes.

## Introduction

Nitrogen is an essential element for all living organisms, particularly for legume crops producing seeds of high protein content. In nature, legumes are mostly able to self-supply nitrogen due to the establishment of symbiosis with rhizobia in root nodules for biological nitrogen fixation (BNF), which contributes to sustainable agriculture (Peoples et al., 1995). During the symbiosis, nitrogen from the atmosphere is converted into ammonia by rhizobia in root nodules, as nutrients to the plants (Wilson and Burris, 1947). In return, rhizobia take carbon from the host plants for their energy.

During the interaction between legumes and rhizobia, rhizobia first enter host plant roots either intracellularly *via* root hair or intercellularly *via* cracks on root surface (Madsen et al., 2010). Nodules are then initiated in the root cortex beneath the rhizobial infection site as the infection of rhizobia proceeds (Oldroyd, 2013). Rhizobia infect plant cells in the nodule primordia, where they differentiate into bacteroid, a form of rhizobia at nitrogen-fixing state. Up to date nearly two hundred genes required for the symbiosis process have been identified and characterized, mainly in two model legumes, *Lotus japonicus* and *Medicago truncatula* (Roy et al., 2020). The molecular signaling processes initiate when the legumes release flavonoids to attract rhizobia. In response, rhizobia secrete nodulation (Nod) factors (NFs), lipochitooligosaccharide (LCOs) signaling molecules, which can be recognized by NF receptor 1 (NFR1) and NFR5 based on the studies in *L. japonicus* (Limpens, 2003; E. B. Madsen et al., 2003). The interaction between NFR1/5 and NFs stimulates symbiotic calcium oscillations, which are further decoded by Ca^2+^/calmodulin-dependent protein kinase (CCaMK) and CYCLOPS (Levy et al., 2004; Yano et al., 2008). The output of the symbiotic signal is further transmitted by the GRAS transcriptional factors (TFs), nodulation signaling pathway 1 (NSP1) and NSP2 (Oldroyd and Long, 2003; Kalo, 2005; Arrighi et al., 2006) as an NSP1- NSP2 complex to induce Nodule Inception (*NIN)* and required for Nodulation 1 (*ERN1)* genes for nodule organogenesis process (Stracke et al., 2002; Marsh et al., 2007).

Cultivated peanut (*Arachis hypogaea* L.) is an important legume crop grown worldwide. It is an allotetraploid (2n= 4x = 40, AABB) with a genome size of ~ 2.7 Gb (Bertioli et al., 2019). As a legume species, cultivated peanut plants can establish symbiosis with *Bradyrhizobia*, a genus of soil-born slow-growing bacteria, and produce nodules regularly. Mutants of non-nodulating (Nod–) peanuts were first reported in progeny derived from the cross between two normally nodulating (Nod+) lines, PI 262090 and UF 487A-4-1-2 (Gorbet and Burton, 1979). A recent effort of a forward genetics approach uncovered that natural nucleotide polymorphisms at a pair of *NSP2* homoeologs caused the Nod– mutants in cultivated peanuts (Peng et al., 2021). This pair of homoeologous *NSP2* genes are located on chromosomes 08 and 17 of two subgenomes A and B, and thus were named *AhNSP2-A08* (*N_a_*) and *AhNSP2-B07* (*N_b_*), respectively. The natural mutant allele *n_a_* is a single nucleotide polymorphism (SNP) from cytosine to thymidine at the 673th nucleotide in the coding region, which leads to a premature stop codon that reduces the polypeptide size from 513 to 224 amino acids (Peng et al., 2021). The natural mutant allele *n_b_* is a single nucleotide deletion of the cytosine at the 119^th^ nucleotide in the coding region, which causes a reading frame shift and leads to a premature stop codon, reducing the polypeptide size from 516 to 113 amino acids (Peng et al., 2021). Predictably, the protein encoding sequences of both mutated alleles *n_a_* and *n_b_* miss all or most of the functional domains of the GRAS TF.

In our previous research (Peng et al., 2021), crossing of the Nod+ PI 262090 and UF 487A-4-1-2 with the confirmed genotypes of *N_a_N_a_n_b_n_b_* and *n_a_n_a_N_b_N_b_* produce a Nod+ F_1_ (*N_a_n_a_N_b_n_b_*), the F_2_ selfing population derived from which segregates the Nod– phenotype with the homozygous recessive alleles at both loci (*n_a_n_a_n_b_n_b_*), confirming the roles of both *N_a_* and *N_b_* in nodule production. Our further analysis revealed that the segregation of the nodulation phenotype follows the Mendelian inheritance with a 3:1 ratio of Nod+: Nod– at the *N_a_* locus in a selfing population of a heterozygous plant with the genotype of *N_a_n_a_n_b_n_b_*. The plants with the genotype *N_a__n_b_n_b_* are Nod+, and the plants with the *n_a_n_a_n_b_n_b_* genotype are Nod–. By contrast, the phenotypic segregation ratio of Nod+ to Nod– varied from 5:3 to 1:1 at the *N_b_* gene locus in the selfing population of a heterozygous plant with the genotype of *n_a_n_a_N_b_n_b_*, violating a Mendelian 3:1 ratio. We further confirmed that the phenotype of the heterozygous lines with the genotype of *n_a_n_a_N_b_n_b_* can be either Nod+ or Nod– (Peng et al., 2021), suggesting that some other mechanisms are underlying in regulation of the nodule phenotype at the *N_b_* locus.

In this study, to understand the genetic mechanisms of the *AhNSP2-B07* gene in controlling peanut nodulation, we analyzed the segregating ratios of peanut nodulation at the *N_b_* locus in the field, allelic expression of *N_b_* in peanut roots and gametic tissues, and DNA methylation at the *N_b_* locus. Our results revealed that the expression of *AhNSP2-B07* is paternally expressed or imprinted in the peanut genome showing parent-of-origin effects. Although the bisulfite sequencing of the promoter and coding regions of *AhNSP2-B07* revealed no significant differences in the methylation level between ovary and pollen, it is suspected that either the tissues we sampled didn’t resolve to detect the gametic methylation variations or the imprinting is not due to DNA methylation. To our knowledge, this is the first functional embryonic imprinted gene identified in crop species.

## Materials and methods

### Plant materials and segregating populations in the field

**Figure 1:**
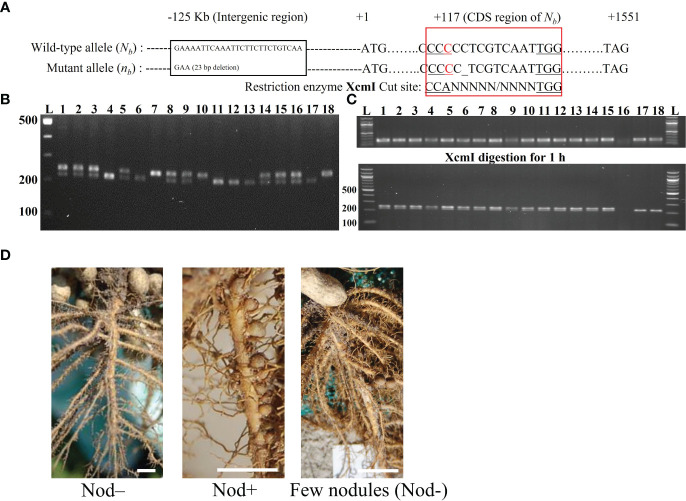
Genotyping strategies at the *N_b_* locus using different markers and phenotyping of nodulation. **(A)** Location of the INDEL marker and the CAPS marker. The INDEL marker was located at 125 Kb upstream of *AhNSP2-B07* (*N_b_*), with a 23 bp deletion in the mutant allele. For the CAPS marker, a “C” to “A” mismatch in the forward primer was introduced in the 117^th^ position of the CDS region in *AhNSP2-B07* to create the cutting site of XcmI for the mutant allele. “_” indicates the 1 bp deletion of cytosine in *n_b_* allele. The wild-type allele was not recognized by XcmI due to the one extra nucleotide. **(B)** Example of genotyping using the AhB07NSP2_INDEL marker. The genotypes of individual plants (Lane 1-18) were indicated as *N_b_N_b_*: 240 bp (one upper band); *N_b_n_b_*: 217 bp and 240 bp (two bands); *n_b_n_b_*: 217 bp (one lower band). L: 100 bp DNA ladder. **(C)** Example of genotyping individual plants (Lane 1-18) using the AhB07NSP2_CAPS marker. Top panel, before digestion (246 bp). Bottom panel, after digestion, *N_b_N_b_*: 246 bp; *N_b_n_b_*: 226 bp and 246 bp; *n_b_n_b_*: 226 bp. L: 100 bp DNA ladder. **(D)** Nodulation phenotypes. Plants with a few big nodules were classified as Nod–.

Peanut lines derived from the cross between Nod– E4 (*n_a_n_a_n_b_n_b_*) and Nod+ E5 (*n_a_n_a_N_b_N_b_*) were used in this study. E4 and E5 are sister inbred lines derived from parental lines UF 487A (Nod+) and PI 262090 (Nod+) (Peng et al., 2018), both of which are Virginia botanical types. The F_5_, F_6_, and F_7_ generations of heterozygous *N_b_n_b_* Nod+ lines from a cross between E4 and E5 were planted in the year 2018, 2019, and 2020, respectively. Peanut seeds were planted from late April to early May and harvested in September every year at the University of Florida Plant Science Research and Education Unit (PSREU, Citra, FL). Commercial inoculum Optimize (Monsanto, St. Louis, MO) was applied to promote nodulation in the field when seeds were planted. The nodulation phenotype was recorded with visual assessment during harvest. Peanut plants were dug out and the root nodule on the whole root system of each plant was scored as a qualitative trait. The phenotypes were recorded as Nod+ if the plants nodulated normally or Nod– if the plants failed to produce any nodule. In a few incidences, peanut root had only a few unusual big nodules, which exhibited aboveground symptoms of nitrogen depletion, were classified as Nod– as well (**Figure 1D**).

### Inoculation and sampling in the greenhouse

The *Bradyrhizobia* strain Lb8 (Paudel et al., 2020) was grown at 28°C in 30 ml YEM media with 20 µg/mL chloramphenicol in a 50 ml Falcon tube on a rotary shaker at 50 rpm for one week. When the optical density (OD) at 600 nm of the Lb8 culture reached 0.8, the cells were collected by centrifuge, washed, and adjusted to OD_600_ of 0.15 as inoculum.

Peanut seeds collected from heterozygous plants (*N_b_n_b_*) were sterilized with 0.1% Mercury chloride for seven minutes, washed twice with sterile water, soaked in Abound fungicide (Syngenta, Wilmington, DE) for 5 mins, washed three times with sterile water, and then transferred into the germination box and incubated at 28°C under dark in the growth chamber. When the lateral roots started emerging around one week after seed germination, peanut roots were dip-inoculated with Lb8 inoculum. Seedlings that were dipped into the water are treated as negative controls. After inoculation, the seedlings were incubated in the germination box for two days before being transferred into the soil.

The primary roots of the seedlings at 2 days post-inoculation (dpi), 5 dpi, and 14 dpi were excised and snap-frozen with liquid nitrogen for RNA extraction and allelic expression examination. Biological replicates at each time point were obtained solely from seedlings that were of comparable height and size. The total RNA of the root samples was extracted using the Direct-zol RNA extraction kit (ZYMO research, Irvine, CA) following the manufacturer’s instructions. Three micrograms of the total RNA from each sample were then reverse-transcribed into cDNA using SuperScript™ III First-Strand Synthesis System (Invitrogen, Waltham, MA). The seedlings with excised roots were re-planted in the soil pre-mixed with the *Bradyrhizobia* inoculum for phenotyping. The plants were dug out one month later after sampling to check the presence of nodules for phenotyping.

### Plant genotyping and allelic expression

Young leaves were collected from each plant either in the field or in the growth chamber for DNA extraction using MagJET magnetic bead (Thermo Fisher, Waltham, MA) following the protocol. A 23 bp deletion in the mutant allele (E4) located ~125 Kb apart from the *AhNSP2-B07* gene was identified from the sequences generated from a QTL-seq experiment (Peng et al., 2021), which was used as an intergenic INDEL marker (AhB07NSP2_INDEL, **Figure 1A**) for genotyping the segregating population in the field (**Figure 1B**). *Chi*-square tests were conducted to check the fitness of the phenotypic and genotypic segregation ratios.

To distinguish the single nucleotide deletion of the mutant allele *n_b_* from *N_b_*, a CAPS marker, AhB07NSP2_CAPS (**Figure 1A**) was designed with indCAPS (http://indcaps.kieber.cloudapps.unc.edu/) using the sequences surrounding the mutation site. The restriction enzyme *XcmI* (New England Biolabs, Ipswich, MA) was chosen to digest the PCR fragments with the maximal length difference between the *N_b_* and *n_b_* alleles. One artificial mismatch was introduced in the forward primer to create the recognition site of *XcmI* in the mutant genotype (*n_b_*). First, a touchdown PCR program was used with the annealing temperature dropping from 65°C to 55°C and with an extension time of 10 s. PCR products using the root cDNA as the template were examined on a 1% agarose gel to verify the size of the product. Next, 4 µl of the PCR product was digested with *XcmI* enzyme at 37°C for 1 hour following the manufacturer’s instruction. The digested product was visualized by gel electrophoresis with a 2% agarose gel for genotype calling (**Figure 1C**). The PCR amplicons were further validated using Sanger sequencing (Genewiz, South Plainfield, NJ) as well.

### qRT-PCR analysis with gametic cells

Three biological replicates of plants of Nod+ *N_b_N_b_*, Nod– *n_b_n_b_*, Nod+ *N_b_n_b_*, and Nod– *N_b_n_b_* were selected in the field for flower collection. Unopened flower buds and fresh flower blooms were collected from each plant and kept separately in RNAlater™ Stabilization Solution (Thermo Fisher, Waltham, MA) for ovary and pollen dissection, respectively (**Supplementary Figure S1**).

Total RNA of ovary and pollen samples dissected from peanut flowers were extracted with RNeasy Plant Mini Kit (Qiagen, Germantown, MD) following the manufacturer’s protocol. Total RNA integrity was examined on a 1% agarose gel by electrophoresis. The quantity was checked using NanoDrop and Qubit RNA broad range (Thermo Fisher, Waltham, MA). Potential residual genomic DNA was further digested by DNA-free™ DNA Removal Kit and Dynabeads™ mRNA Purification Kit (Thermo Fisher, Waltham, MA). One microgram of total RNA from the ovary and pollen samples was respectively used for cDNA synthesis by SuperScript™ III First-Strand Synthesis System from the RT-PCR kit (Invitrogen, Waltham, MA).

qRT-PCR for *AhNSP2-B07* was performed by using primer AhB07NSP2_q (**Supplementary Table 1**) with PowerSYBR Green PCR Master Mix (Thermo Fisher, Waltham, MA) running on QuantStudio™ 6 Flex Real-Time PCR System (Thermo Fisher, Waltham, MA), with three technical replicates using the *AhUBI* gene as the internal control (Morgante et al., 2011). Intergenic marker AhB07NSP2_INDEL (**Supplementary Table 1**) was used to confirm the complete removal of gDNA in the cDNA samples. Quantitative analysis of gene expression was performed by using the 2^−ΔΔCT^ method, and one-way or two-way ANOVA was used for statistical analysis.

### Bisulfite sequencing

A total of 12 peanut plants (3 biological replicates of Nod+ *N_b_N_b_*, Nod– *n_b_n_b_*, Nod+ *N_b_n_b_*, and Nod– *N_b_n_b_*, respectively) with three types of tissues, including ovary and pollen, were collected in the same way as the methods described above and used for DNA extraction. Peanut pegs were collected from the same plants, which were used a somatic-cell control. Bisulfite conversion of genomic DNA samples was performed by using EpiTect Bisulfite Kits (Qiagen, Germantown, MD) according to the manufacturer’s instructions (https://www.zymoresearch.com/pages/bisulfite-beginner-guide). Converted DNA samples were assessed by 2% agarose gel electrophoresis.

**Figure 2:**
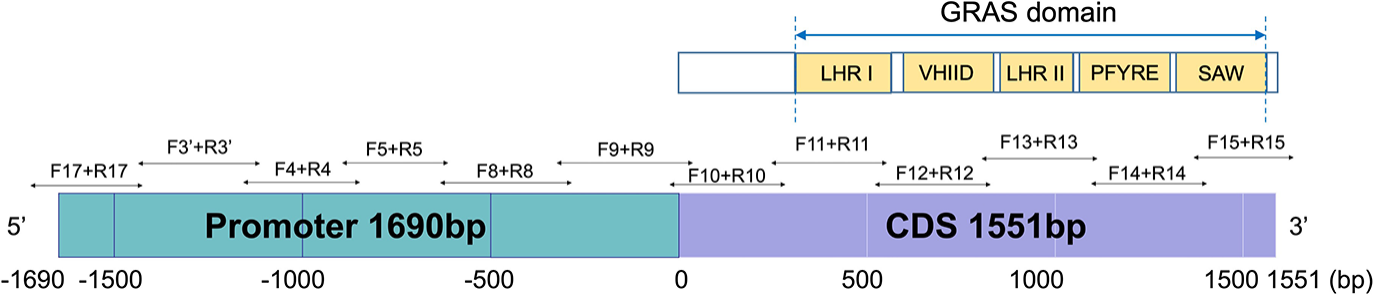
Bisulfite-PCR primer design in the promoter and the full length CDS region of *AhNSP2B07* and schematic representation of the structure and motifs of AhNSP2 GRAS protein.

Primers for bisulfite PCR were designed manually with several considerations: 1) limiting the amplicon size to a range of 300-500 bp, due to the DNA fragmentation during the bisulfite-converting process, 2) avoiding primer sites at any cytosine context due to their unknown methylation status, 3) avoiding non-specific sequence regions with the DNA sequences similarity higher than 98% between *N_a_* and *N_b_* homoeologs, and 4) comparable T_m_ value of the forward and reverse primers. In total, 12 pairs of primers were designed for bisulfite sequencing (**Figure 2**). Primers were designed to cover the region of the whole *N_b_* gene from -1,690 (~the start of promoter region) to +1,551 bp (the end of CDS region) (**Figure 2**), and synthesized by Integrated DNA Technologies, Inc (IDT, Coralville, IW). The primers were primarily designed to be degenerate as C/T at the C sites if the C sites could not be avoid. According to the amplicon sequencing results, specific primers were further designed. Bisulfite-converted and unconverted genomic DNA samples were used as templates to test the specific primers. Primers only amplify the bisulfite converted but not the unconverted DNA sample were used in the experiments to ensure optimal amplification only from bisulfite-converted DNA samples. All primer sequences for bisulfite sequencing are listed in **Supplementary Table 1**.

PCR reactions were performed using EpiMark^®^ Hot Start Taq DNA Polymerase (New England BioLabs, Ipswich, MA) and GoTaq^®^ Master Mixes (Promega Corporation, Madison, WI). The PCR products were validated on a 2% agarose gel. Faint PCR bands were extracted from agarose gel and purified using Zymoclean Gel DNA Recovery Kit (Zymo Research, Irvine, CA). The purified DNA fragments were then used as templates to perform the second round of PCR amplification. All PCR products were sent for Sanger sequencing (Genewiz, South Plainfield, NJ) with either forward or reverse primers.

### Bisulfite-sequencing data analysis

The SnapGene software (Insightful Science, San Diego, CA) was used to align and compare the Sanger sequences with the reference DNA sequence. All the successful Sanger sequencing reads were firstly aligned to the *N_a_* and *N_b_* sequences to make sure that the reads were specifically amplified from the *N_b_* gene. According to the *N_b_* gene sequence, cytosine sequence contexts were classified as CG (M1), CHG (M2, H = A, T, or C), and CHH (M3) contexts. Methylation status in different contexts was recorded manually for each amplicon, separately. The methylation status was recorded as “1” if completely methylated showing a clear “C” peak, or as “0” if unmethylated showing a clear “T” peak on the chromatograph. If both C and T peaks were present (overlapping C and T peaks), “0.5” was recorded to indicate partial methylation. Missing data was labelled as “”.

The methylation ratios of individual cytosine sites were recorded for ovary, pollen, and peg tissues, respectively. The average methylation ratio in the three methylation contexts of three types of tissue was analyzed by two-way ANOVA. A logistic regression method (Position/Total Number of Observations) was used to analyze the proportion of Plant IDs with a positive “1” score for methylation and calculated both overall P-values as well as contrast P-values comparing ovary to pollen tissues. Other statistical analyses, including Least squares means, Odds-ratios, and the approximate 95% confidence interval, were calculated as well.

## Results

### The ratio of Nod+ to Nod- of *N_b_n_b_* lines is distorted from 3:1

A total of 787, 316, and 119 peanut plants derived from *N_b_n_b_* lines were phenotyped for nodulation over three consecutive years as F_5_, F_6_ and F_7_ populations. The phenotypic segregation ratio in each population was distorted from Mendelian 3:1 ratio (**Table 1**). For example, out of the 787 F_5_ plants, 469 and 318 are Nod+ and Nod-, respectively, which is significantly distorted from either 3:1 or 1:1 (**Table 1**). Next, we examined the genotypes of these three populations at the *N_b_* locus. Genotyping the randomly selected 362, 34, and 109 individuals from F_5_, F_6_, and F_7_ populations showed that the segregation ratio of genotypes *N_b_N_b_*: *N_b_n_b_*: *n_b_n_b_* fitted the 1:2:1 ratio in all three populations (**Table 1**). The phenotypes of plants with *N_b_N_b_* and *n_b_n_b_* genotypes were Nod+ and Nod– (**Figure 1D**), respectively, with no exceptions. However, phenotypes of the *N_b_n_b_* plants were either Nod+ or Nod–, which caused the phenotypic segregation ratio to be distorted from the 3:1 ratio. A small portion of plants (~5%) with few unusual big nodules were also observed with *N_b_n_b_* genotype (**Figure 1D**), which were classified as Nod– as they exhibited deficit of nodulation and nitrogen deficiency phenotype.

**Table 1:** Phenotypes and genotypes segregating in F_5_, F_6_, and F_7_ populations derived from heterozygous (*N_b_n_b_*) plants in the field in 2018, 2019, and 2020, respectively.

	Phenotypes						Genotypes				
Generation/Year	Nod+	Nod–	Nod+:Nod–	χ^2^ of 3:1		χ^2^ of 1:1	*N_b_N_b_*	*N_b_n_b_*	*n_b_n_b_*	*N_b__: n_b_n_b_*	χ^2^ of 1:2:1
F_5_, 2018	469	318	1.47:1	1.84E-23		6.71E-05	96	185	81	3.46:1	0.49
F_6_, 2019	163	153	1.07:1	7.00E-22		0.09	6	20	8	3.25:1	0.52
F_7_, 2020	76	43	1.77:1	0.01		0.10	27	51	31	2.51:1	0.69

A portion of plants instead of the whole population was randomly selected and genotyped each year. Thus, the total number of genotyped plants was smaller than the total number of the whole population in each generation.

### Monoallelic expression of *AhNSP2-B07* in peanut roots with the heterozygous genotype (*N_b_n_b_*)

**Figure 3:**
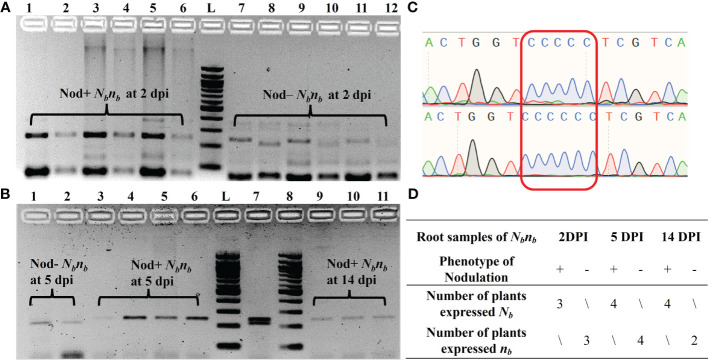
Monoallelic expression of *AhNSP2-B07* in root tissues of *N_b_n_b_* at 2 dpi, 5 dpi, and 14 dpi. **(A)** PCR of root cDNA samples at 2 dpi. Lane 1-6: biological replicates of Nod+ *N_b_n_b_*, in which lanes 2, 4, and 6 were *XcmI*-digested PCR products of lanes 1, 3, and 5, respectively. Lane 7-12: biological replicates of Nod– *N_b_n_b_*, in which lanes 8, 10, and 12 were *XcmI*-digested PCR products of lanes 7, 9, and 11, respectively. **(B)** PCR of root cDNA samples at 5 and 14 dpi after *XcmI* digestion. Lane 1 and 2: Nod– *N_b_n_b_* at 5 dpi. Lane 3-6: Nod+ *N_b_n_b_* at 5 dpi. Lane 7: gDNA of *N_b_n_b_* as a control. Lane 8-11: Nod+ *N_b_n_b_* at 14 dpi. **(C)** Sanger sequencing of the PCR products amplified from *N_b_n_b_* root cDNA. Two representative samples from Nod– *N_b_n_b_* (top) and Nod+ *N_b_n_b_* (bottom), respectively. **(D)** Summary of all monoallelic expressions at different time points. L: 100 bp DNA ladder.

AhB07NSP2_CAPS was used to determine the allelic expression of the *N_b_* gene in the root samples of *N_b_n_b_* plants (**Figure 3**). Owing to variability in germination time and rate, as well as the segregation at the *N_b_* locus, the number of biological replicates of Nod+ and Nod– *N_b_n_b_* plants at each time point is limited. For root samples at 2 dpi, the *N_b_* gene was exclusively expressed in all three Nod+ *N_b_n_b_* plants, while the mutant allele *n_b_* was solely expressed in the three Nod– *N_b_n_b_* plants, exhibiting monoallelic expression (**Figure 3A**). The same monoallelic gene expression was also observed in the root samples at 5 dpi and 14 dpi (**Figure 3B**). Specifically, at 5 dpi, the *n_b_* allele was exclusively expressed in two randomly selected Nod– *N_b_n_b_* root samples, while *N_b_* was expressed in four Nod+ *N_b_n_b_* roots (**Figure 3B**). At 14 dpi, only *N_b_* was found to be expressed in three Nod+ *N_b_n_b_* root samples (**Figure 3B**). PCR amplification was very weak from Nod– *N_b_n_b_* at 14 dpi, thus no results were generated. The PCR amplicons from the cDNA of *N_b_n_b_* root samples were further confirmed by Sanger Sequencing (**Figure 3C**). The single clear peaks of the chromatogram indicated that all the Nod– *N_b_n_b_* only expressed the *n_b_* allele, while Nod+ *N_b_n_b_* only expressed the *N_b_* allele (**Figure 3D**). The amplification from cDNA samples of peanut leaves was barely visible on the gel, suggesting low expression of the *N_b_* gene in peanut leaves (Data not shown).

### The expression of *AhNSP2-B07* is higher in pollen than ovary

**Figure 4:**
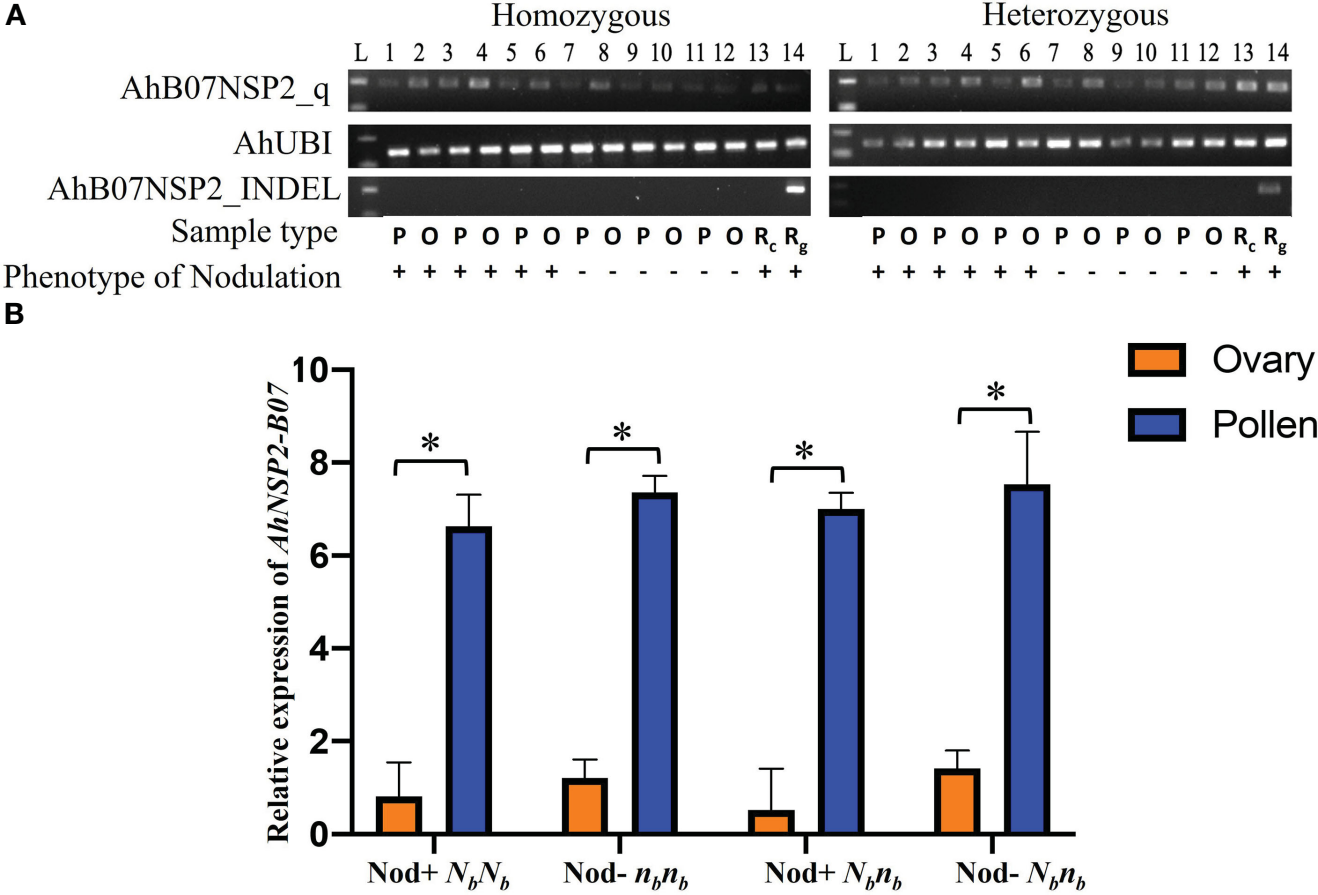
Comparison of expression of *N_b_* reveals significantly higher expression in pollen. **(A)** PCR products of AhB07NSP2_qw1, AhUBI, and AhB07NSP2_INDEL. For homozygous samples, Lane 1-6: *N_b_N_b_*; Lane 7-12: *n_b_n_b_*. For heterozygous samples, Lane 1-6: Nod+ *N_b_n_b_*; Lane 7-12: Nod– *N_b_n_b_.* L: 100 bp DNA ladder. P: pollen. O: ovary. R_c_: root cDNA. R_g_: root gDNA. **(B)** Relative expression of *AhNSP2-B07* across different genotypes and phenotypes. Ovary and pollen samples were collected from homozygous Nod– *n_b_n_b_*, Nod+ *N_b_N_b_*, and heterozygous *N_b_n_b_* with Nod+ and Nod– phenotypes. The p-value was conducted by student’s *t* test. *p < 0.05. The error bars represent the standard deviation of each sample.

The monoallelic expression of *AhNSP2-B07* led us to ask whether *AhNSP2-B07* is imprinted between female and male alleles. To test this, ovary and pollen tissues were dissected from Nod+ heterozygotes (*n_a_n_a_N_b_n_b_*), Nod– heterozygotes (*n_a_n_a_N_b_n_b_*), Nod– homozygotes (*n_a_n_a_n_b_n_b_*), and Nod+ homozygotes (*n_a_n_a_N_b_N_b_*) for qRT-PCR analysis at the *N_b_* locus. As expected, no amplification was detected using AhB07NSP2_INDEL (**Figure 4A**), which targets an intergenic region. All the samples got successful amplification with the housekeeping gene *AhUBI* (**Figure 4A**). The qRT-PCR results showed that the expression levels of the *N_b_* gene were significantly higher the in pollen (*p*<0.0001) than that in the ovary (**Figure 4B**), regardless of the nodulation phenotype or genotype of the plants at the *N_b_* locus. This result demonstrated that the *N_b_* gene’s expression was significantly suppressed in the female gametes of all peanut plants, suggesting that *N_b_* is likely an imprinted gene.

### No significant difference in DNA methylation at *N_b_* between pollen and ovary

**Figure 5:**
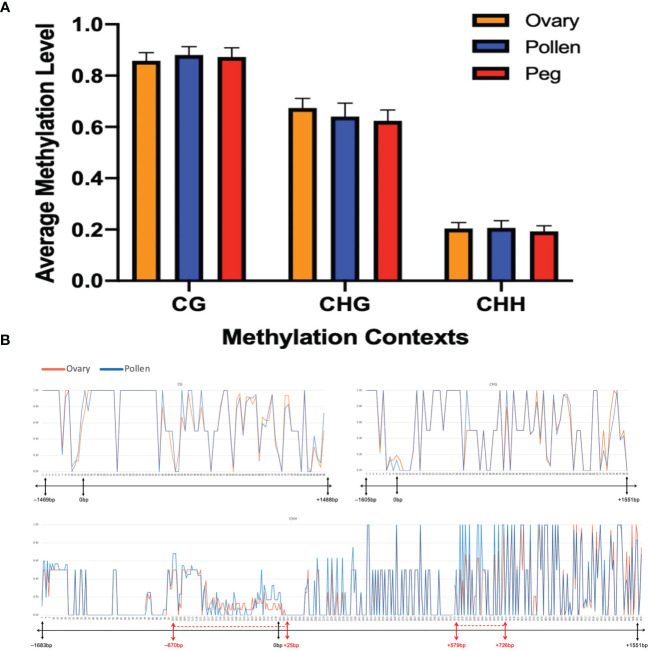
No significant difference in methylation level at *N_b_* between ovary and pollen. **(A)** Average methylation level in CG, CHG, and CHH contexts in the three plant tissues (*p*=0.1531). **(B)** Distribution of methylation levels in CG, CHG, and CHH contexts across the *N_b_* gene between ovary and pollen. The X-axis represents the position of the methylation sites in the detected sequence. The bar under each X-axis shows the physical location of the promoter and CDS of the *AhNSP2-B07* gene. Regions showing different methylation levels between ovary and pollen are indicated with red dotted lines on the bar.

Genomic imprinting is an epigenetic phenomenon that involves DNA cytosine methylation (Rodrigues and Zilberman, 2015). To further understand whether the biased expression of the *N_b_* gene in peanut gametes is caused by the difference in DNA methylation between female and male, bisulfite PCR and subsequent sequencing were conducted on the *N_b_* gene including the promoter region from gametic tissues of Nod+ heterozygotes (*n_a_n_a_N_b_n_b_*), Nod– heterozygotes (*n_a_n_a_N_b_n_b_*), Nod– homozygotes (*n_a_n_a_n_b_n_b_*), and Nod+ homozygotes (*n_a_n_a_N_b_N_b_*). Out of 432 PCR reactions, 370 (87%) produced specific amplicons with successful Sanger sequence reads (**Table 2**). The rest were either not amplified, or amplified un-specifically, or failed to be sequenced, which were all treated as missing data. All the 370 reads contained cytosines of methylated, unmethylated, or partially methylated, which basically indicated the effectiveness of bisulfite conversion. Across the full *N_b_* gene sequence including promoter and CDS regions (3,241 bp), 633/642 cytosine sites (98.6%) were covered by the sequencing data, including 88 CG, 78 CHG, and 467 CHH sites. Among the 633 cytosine sites, 200 were unmethylated across all the samples tested. Of the rest methylated sites, the three different types of tissue had no significantly different methylation levels in the overall *N_b_* gene sequence (*p*=0.1531) in all three cytosine contexts. The CG context had the highest methylation levels (0.82 ~ 0.93) followed by CHG (0.52 ~ 0.72), and CHH (0.18 ~ 0.25) (**Figure 5A**).

**Table 2:** The average ratio of successfully sequenced cytosine site in peanuts.

Genotypes	Phenotypes	Number of plants tested	Tissue	Average Ratio of Sequenced C Sites
*n_a_n_a_N_b_N_b_*	Nod+	3	Ovary	81.15%
*n_a_n_a_n_b_n_b_*	Nod–	3	Ovary	82.04%
*n_a_n_a_N_b_n_b_*	Nod+	3	Ovary	75.91%
*n_a_n_a_N_b_n_b_*	Nod–	3	Ovary	78.35%
*n_a_n_a_N_b_N_b_*	Nod+	3	Pollen	57.01%
*n_a_n_a_n_b_n_b_*	Nod–	3	Pollen	63.80%
*n_a_n_a_N_b_n_b_*	Nod+	3	Pollen	65.20%
*n_a_n_a_N_b_n_b_*	Nod–	3	Pollen	59.10%
*n_a_n_a_N_b_N_b_*	Nod+	3	Peg	79.75%
*n_a_n_a_n_b_n_b_*	Nod–	3	Peg	66.72%
*n_a_n_a_N_b_n_b_*	Nod+	3	Peg	66.46%
*n_a_n_a_N_b_n_b_*	Nod–	3	Peg	81.83%

At a few regions, such as from –670 bp to 25 bp (mostly in the promoter region), 579 bp to 726 bp, etc., slightly different methylation level was observed between the two gametic tissues (**Figure 5B**). When look at each individual cytosine site, 269 out of the 633 sequenced cytosine sites, including 200 unmethylated and 69 methylated sites, showed the same methylation rate in all tested samples. For the rest 364 sites, no significant differences of methylation level were identified between ovary and pollen samples.

## Discussion

### Expression bias of homoeologs in polyploids

Polyploidy, or whole genome duplication, creates novel phenotypes that enable the polyploids to better adapt to environmental changes, which is critical for crop evolution and crop domestication (Chen et al., 2007; Yoo et al., 2014). As a result of polyploidization, duplicated genes from two subgenomes known as homoeologs, may share the same functions and expression patterns or have different functions. Several potential fates could occur for homoeologs after whole genome duplication. For example, both genes keep the original function, one copy gets silenced and becomes pseudogene, or the genes diversify with different functions or expression patterns (Lynch and Force, 2000). Both natural polyploids and synthetic polyploids have been investigated for homoeolog expression bias at the whole genome level. Allotetraploid cotton (*Gossypium hirsutum*) (AADD) is one of the most widely cultivated crops from hybridization between *G. arboreum* (A2) and *G. raimondii* (D5), with 15.90–37.96% of genes showed different expression biases towards A or D subgenomes in fibers among different cultivated cotton cultivars (Mei et al., 2021). In another study, *Raphanobrassica* (RRCC), which was artificially synthesized from *Raphanus sativus* (RR) and *Brassica oleracea* (CC) showed genome-wide unbalanced biased expression bias towards *B. oleracea* (Zhang et al., 2021). In *Arabidopsis suecica*, more sequence deletions were detected in the less-expressed subgenome (Chang et al., 2010; Garsmeur et al., 2014), which might lead to biased expression of the homoeologs.

While most of the species in the genus *Arachis* are diploid, *A. hypogaea* is an allotetraploid species, which is most likely derived from the hybridization between two wild diploid Arachis ancestors, followed by polyploidization (Husted, 1936; Burris and Roberts, 1993; Kochert et al., 1996). Overall, the total number of biased expressed homoeologs towards the A subgenome was similar to the number towards the B subgenome (Bertioli et al., 2019). However, this difference is significant in some specific tissues, such as pericarp, perianth, root, and peg, which have significantly higher number of highly expressed genes in the B subgenome than in the A subgenome (Bertioli et al., 2019).

For the *NSP2* homoeologs in peanuts, the segregation of *AhNSP2-A08* fits the Mendelian segregation ratio, while the segregation of *AhNSP2-B07* does not. Based on the survey of the mutation rate of the two *NSP2* homoeologs in a US mini core collection, *AhNSP2-A08* has a much lower loss of function mutation rate than *AhNSP2-B07* (Peng et al., 2021), which may suggest that *AhNSP2-A08* has undergone stronger purifying selection during evolution while *AhNSP2-B07* is gradually losing its function with a higher frequency of mutation rates. Moreover, it has been revealed that *AhNSP2-A08* was highly expressed only in roots, whereas *AhNSP2-B07* was highly expressed in both roots and flowers (Peng et al., 2021). This indicated that *AhNSP2-B07* may be related to the reproductive process.

### Parental effect controlling *AhNSP2-B07*

In a previous study (Gallo-Meagher et al., 2001), reciprocal crosses were made between Nod– M4-2 (*n_a_n_a_n_b_n_b_*) and Nod+ PI 262090 (*n_a_n_a_N_b_N_b_*), the parental lines of E4 and E5 used in this study. All 30 F_1_ progeny were Nod+ with uncountable nodules only when the wild-type allele *N_b_* was from the male parent (**Table 3**). However, when the wild-type allele *N_b_* was inherited from the female parent, most of the F_1_ progeny (32 out of 33) were Nod– showing non-nodulation or only few unusual big nodules (**Table 3**). The dramatic difference in F_1_ phenotypes between reciprocal crosses suggested a parent-of-origin effect (Gallo-Meagher et al., 2001) at *N_b_*. For the reciprocal crosses between Nod+ UF487A (*N_a_N_a_n_b_n_b_*) and Nod- M4-2 (*n_a_n_a_n_b_n_b_*), all F_1_ progeny (32 and 26), except one, were Nod+, suggesting that *N_a_* had no imprinting effect. Therefore, we hypothesized that the allele from female gametes in peanuts at the *N_b_* locus is inhibited or imprinted with little or no expression, whereas the allele from male gametes at *N_b_* can express normally in the offspring (**Table 4**), which was further validated by qRT-PCR on gametic tissue (**Figure 4**). According to this hypothesis, an equal number of Nod+ and Nod– *N_b_n_b_* offspring should be produced from the selfing population of *N_b_n_b_* plants, and thus a 1 Nod+:1 Nod– segregating ratio was expected to be observed in the field. In reality, the segregating ratio of Nod+: Nod– fell within the range of 5:3 to 1:1 (Peng et al., 2021). Considering the Nod– plants have a relatively lower survival rate in the field due to nitrogen depletion, as starter nitrogen fertilizer was only applied during planting time. The practice of limiting nitrogen fertilizer maximized the phenotypic differences between Nod+ and Nod– peanut plants in the field, which also reduced the survival rate of low vigor Nod– plants inevitably. Therefore, the segregating ratio observed in the field during harvest is very likely shifted from the 1:1 ratio to some extent.

**Table 3:** Phenotypes of F1 progeny of reciprocal crosses between different genotypes of nodulating and non-nodulating plants (Adapted from Gallo-Meagher et al., 2001).

Cross		Genotypes	Phenotypes of F_1_		
Female	Male		Nod+	Few nodules	Nod–
UF 487A	M4-2	*N_a_N_a_ n_b_n_b_* × *n_a_n_a_ n_b_n_b_*	32	0	1
M4-2	UF 487A	*n_a_n_a_ n_b_n_b_* × *N_a_N_a_ n_b_n_b_*	26	0	0
PI262090	M4-2	*n_a_n_a_ N_b_N_b_* × *n_a_n_a_ n_b_n_b_*	1	24	8
M4-2	PI 262090	*n_a_n_a_ n_b_n_b_* × *n_a_n_a_ N_b_N_b_*	30	0	0

**Table 4:** Punnett square showing offspring genotypes of selfing *N_b_n_b_* peanut plants under genomic imprinting.

		Male gametes	
		*N_b_*	*n_b_*
Female gametes	N_b_*	*N_b_* *N_b_ (Nod+)	*N_b_* *n_b_ (Nod–)
	n_b_*	*N_b_n_b_* * (Nod+)	*n_b_* *n_b_ (Nod–)

* Indicates inhibition of the expression of AhNSP2-B07 in the ovary and the marked allele is not expressed. “+” means Nod+ phenotype; “–” means Nod– phenotype.

Based on the qRT-PCR analysis, the expression of *AhNSP2-B07* showed a significantly higher expression in pollen than that in ovary tissues. This result further supports that allele of *AhNSP2-B07* in female gametes is inhibited, which is likely due to genomic imprinting. In very rare cases, the dominant *N_b_* allele was not completely inhibited in the heterozygous *N_b_n_b_* plants, resulting in few unusual big nodules being produced. The heterozygous plants with few big nodules were not sampled and focused as a separate group mainly due to the extreme low occurrence. The inhibited allele derived from female parents remains low or no expression in the offspring and only the allele from male gametes is expressed, which was validated in rhizobial infected roots, where any allele of *AhNSP2-B07* gene inherited from male parents is actively expressing.

### Bisulfite sequencing reveals no significant difference in methylation between ovaries and pollens

Gene imprinting can be caused by methylation and the process starts in gametes, where the allele is imprinted with low or no expression and subsequently remains inactive in the embryo. We compared the methylation levels of the *N_b_* gene spanning a 1690bp promoter region and the full CDS region between the gametic tissues, ovary and pollen. However, no significant differences were detected at all methylation contexts. This result could be due to the technical difficulties in obtaining pure male and female gametic cells or the detected regulatory region of 1690bp is not sufficient. In this study, the ovary and pollen tissues were used, which still contained a significant number of somatic cells, thus adding huge background noise to precisely detect the DNA methylation level difference between the two different gametic cells as a very small proportion of the total cells in the tissues. Therefore, a single-cell epigenomic technology to precisely reveal the methylation variation at the single-cell level might be critical to further investigate the methylation variations between gametic cells (Luo et al., 2020). For example, single-microspore sequencing of maize has been performed to explore methylation reprogramming during the different developmental stages in plant sexual reproduction (Kawashima and Berger, 2014). However, cultivated peanuts have much smaller volume of gametic tissues with a more complicated genome. Thus, the tissue dissection and sequencing approaches need to be specifically optimized to overcome the challenges in peanut research.

Besides DNA methylation, histone methylation such as trimethylation of histone H3 lysine 27 (H3K27me3) catalyzed by Polycomb-Repressive Complex 2 (PRC2) is also associated with imprinting in plants (Köhler and Makarevich, 2006; Wolff et al., 2011). It was reported that the imprinted paternal allele in *Arabidopsis* was mediated by the PcG complex consisting of histone methyltransferase, while no parental DNA methylation asymmetry was detected in the promoter of the *MEDEA* (*MEA*) gene (Wöhrmann et al., 2012). As a future step, the histone modification of the *AhNSP2-B07* gene needs to be investigated.

In summary, this study revealed that the non-Mendelian inheritance of nodulation segregating at *AhNSP2-B07* is due to the inhibition of its expression in female gametes, resulting in further genomic imprinting in the embryo of offspring. This study provided a rare example of a vital symbiosis gene that has parent of origin or maternal imprinting effect in polyploid crops. The monoallelic expression of *N_b_* in root samples explained the phenotypical distortion of Nod+: Nod– from the 3:1 ratio. Combining the reciprocal cross and qRT-PCR results using ovary and pollen, it is confirmed that *N_b_* was maternally imprinted. However, no significant difference of DNA methylation level of the promoter and CDS region of *AhNSP2-B07* was detected between ovary and pollen, and the mechanism underlying the maternal imprinting of this gene remains unclear. Our findings facilitated the understanding of gene regulation in polyploid species, which could be helpful to unveil the evolutionary process after hybridization and polyploidization in cultivated peanuts.

## Data availability statement

The data presented in the study are deposited in the GenBank repository with accession numbers QQ999050 to QQ999064.

## Author contributions

JW conceived the research and designed the experiments. ZZ and YW performed the experiments. ZP helped with primer design and data analysis. ZL helped with the data collection in the field. ZZ and YW drafted the manuscript. MZ provided critical comments on revising the manuscript. All authors contributed to the article and approved the submitted version.

## Supplementary material

Supplementary Figure 1 Tissue collection for ovary and pollen. **(A)**. Bloomed peanut flower for pollen collection **(B)**. Unopened flower bud for ovary collection **(C)**. Stamen **(D)**. Ovary with calyx tube **(E)**. Anther **(F)**. Ovary **(G)**. Pollen grains **(H)**. Ovules. St: stamen. An: anther. Po: pollen. Ov: ovule. Scale bar: a, b = 1 cm. c, d = 1 mm. e, f =0.25 mm. g, h = 50 μm
