# Supplementary figures and images for: Allelic expression of AhNSP2-B07 due to parent of origin affects peanut nodulation
Source: Front Plant Sci. 2023 Jun 22;14:1193465. doi: 10.3389/fpls.2023.1193465 (PMC10325728; doi:10.3389/fpls.2023.1193465)

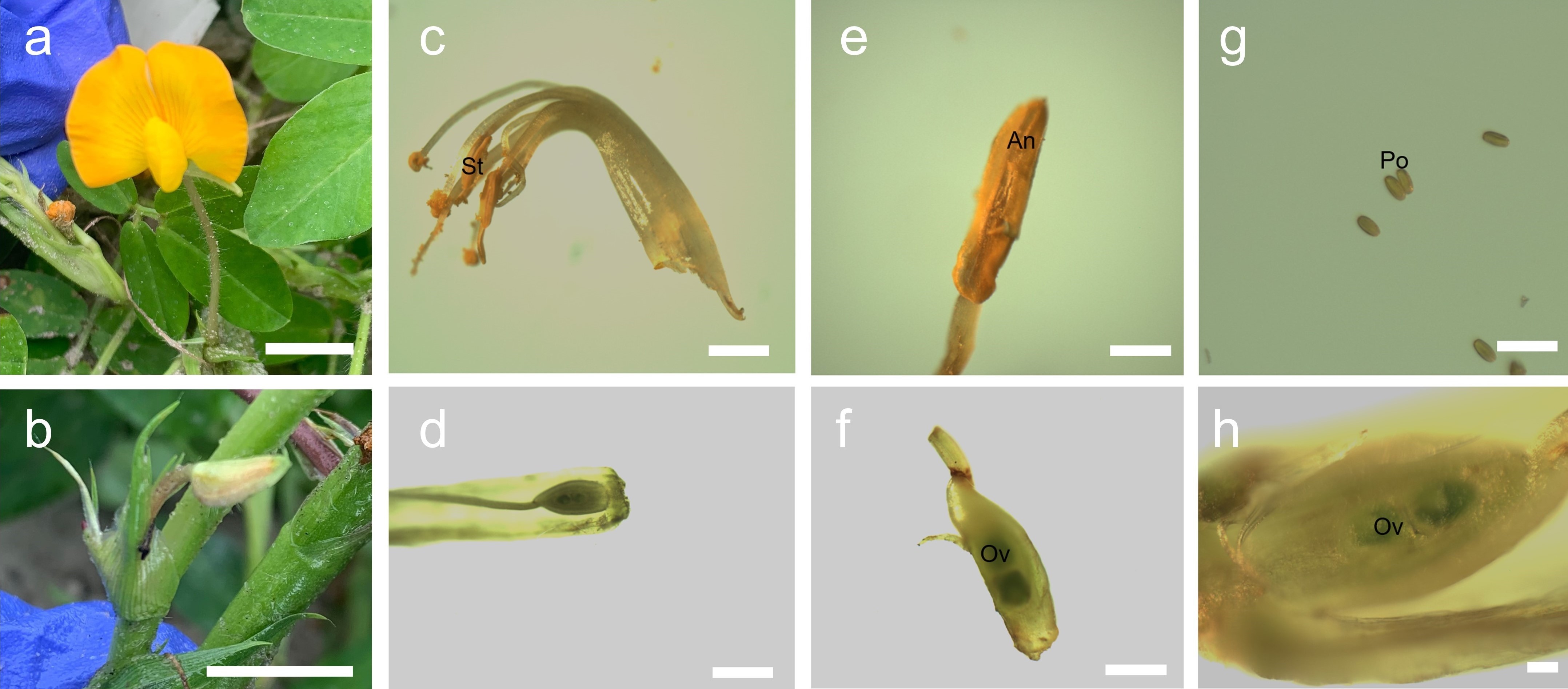

Supplement: Supplementary Figure 1 — Tissue collection for ovary and pollen. (A). Bloomed peanut flower for pollen collection (B). Unopened flower bud for ovary collection (C). Stamen (D). Ovary with calyx tube (E). Anther (F). Ovary (G). Pollen grains (H). Ovules. St: stamen. An: anther. Po: pollen. Ov: ovule. Scale bar: a, b = 1 cm. c, d = 1 mm. e, f =0.25 mm. g, h = 50 μm [file DataSheet_1.zip › Figure S1 .JPEG]
